# Appraisal of jaw swellings in a Nigerian tertiary healthcare facility

**DOI:** 10.4317/jced.51011

**Published:** 2013-02-01

**Authors:** Taye J. Lasisi, Akinyele O. Adisa, Adeola A. Olusanya

**Affiliations:** 1 Lecturer/Consultant. Department of Oral Pathology, University of Ibadan/University College Hospital. Ibadan, Nigeria; 2 Lecturer/Consultant. Department of Oral and maxillofacial surgery, University of Ibadan/University College Hospital. Ibadan, Nigeria

## Abstract

Introduction: The mandible and maxilla can be the site of myriads of lesions that may be categorized as neoplastic, cystic, reactive and infective or inflammatory. Literature reviewing jaw swellings in an amalgamated fashion are uncommon, probably because aetiologies for these swellings are varied. However, to appreciate their relative relationship, it is essential to evaluate the clinico-pathologic profile of jaw swellings. The aim of this appraisal is to describe the array of jaw swellings seen at our hospital from 1990 to 2011, to serve as a reference database. Material and Methods: Biopsy records of all histologically diagnosed cases of jaw swellings seen at the department of Oral Pathology, University College Hospital between January 1990 and December 2011 were retrieved, coded and inputted into SPSS version 20. Data on prevalence, age, sex, site and histological diagnosis were analysed descriptively for each category of jaw swellings. All patients below 16 years were regarded as children. Results: A total of 638 jaw swellings were recorded in the 22-year study period. The Non Odontogenic Tumours (NOT) were the commonest, accounting for 46.2% of all jaw swellings. Odontogenic Tumours (OT) formed 45% of all adult jaw swelling while it formed 25.2% in children and adolescents. Ameloblastoma was the commonest while the most common NOT was ossifying fibroma (OF). Chronic osteomyelitis of the jaws was about 6 times commoner in adult females than males and mostly involved the mandible. The most common malignant jaw swelling was Burkitts’ lymphoma (BL) that was about 7 times more in children than adults. Osteogenic sarcoma was the most common malignancy in adults. Conclusion: Jaw swellings are extensively varied in types and pattern of occurrence. This study has categorized jaw swellings in a simple but comprehensive fashion to allow for easy referencing in local and international data acquisition and epidemiological comparison.

** Key words:**Jaw swellings, odontogenic, Nigeria.

## Introduction

The skeletal framework of the mandible and maxilla can be the site of myriads of lesions that may be categorized as neoplastic, cystic, reactive and infective or inflammatory (1). Lesions developing primarily within the jaws can arise from the dental elements, bone, nerves, ectopic salivary glands, or vascular channels. Classifications of these jaw lesions vary extensively, with no comprehensively accepted system. Some of these classification schemes emphasize the presumed cell of origin of various neoplasms, however as the theories of neoplastic derivations evolve, classification also changes. Indeed as far back as 1958, Scarf and Thompson suggested that the mandible and maxilla could muster a more confusing variety of tumors than any other bone in the body and that any classification scheme may be extremely hazardous (2).

Lesions occurring in the jaws could be either odontogenic or non-odontogenic. Odontogenic tumors (OT) are lesions derived from epithelial, ectomesenchymal and/or mesenchymal components, which are part of the tooth forming apparatus. Non-odontogenic tumors (NOT) can originate from the jawbone and any other mesenchymal tissue; this represents a large heterogeneous group.

Literature reviewing jaw swellings in an amalgamated fashion are uncommon, probably because aetiologies for these swellings are varied. In a five year Kuwaiti (3) review, jaw lesions were divided into three major groups: developmental/inflammatory/reactive lesions (group 1), cystic lesions (group 2), and tumors/ tumor-like lesions (group 3). Groups 2 and 3 were subdivided into OT and NOT. However, to enable realistic practical assumptions and to appreciate a general perspective of these lesions as they relate to each other in occurrence, it is essential to evaluate the clinico-pathologic profile of jaw swellings generally. Thus the aim of this appraisal is to describe the array of all jaw swellings seen at our hospital from 1990 to 2011, which we hope will serve as a reference database regionally and even internationally.

## Material and Methods

Biopsy records of all histologically diagnosed cases of jaw swellings seen at the department of Oral Pathology, University College Hospital between January 1990 and December 2011 were retrieved, coded and inputted into SPSS version 20. All cases of jaw swellings with histological diagnosis were included while cases without histological diagnosis were excluded. Jaw swellings were broadly categorised as benign (OT and NOT) tumors, cysts, infective lesions and malignant neoplasms. OT were classified using the World Health Organisation criteria on histological typing of odontogenic tumors (2005). Infective/inflammatory swellings confirmed by histology were included in this study. Data on prevalence, age, sex, site and histological diagnosis were analysed descriptively for each category of jaw swellings. Data are presented as mean ± SD, percentages and frequencies as appropriate. All patients below 16 years were regarded as children.

## Results

**Figure 1:**
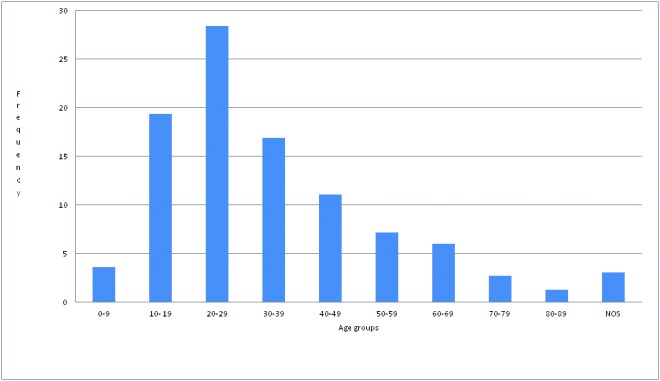
Age distribution of jaw swellings. NOS – not otherwise specified.

**Figure 2:**
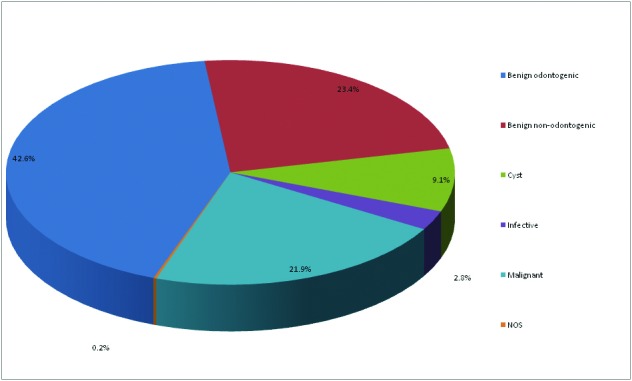
Categories of jaw swellings.

A total of 638 jaw swellings were recorded in the 22-year study period. Jaw swellings were slightly more in males with a male: female ratio of 1.1:1. The overall mean age was 32.36± 17.14 years with a range of 3 to 84 years. Jaw swellings were highest in the 3rd decade of life and consistently decreased till the 9th decade (Fig. 1). Most of the jaw swellings in the maxilla were left sided while those in the mandible were on the right, but overall there were more left sided swellings and mandibular swellings (67.5%) were more than double that in the maxilla (32.2%) ( Table 1). In total the NOT were the commonest, accounting for 46.2% of all jaw swellings (Fig. 2).

**Table 1:**
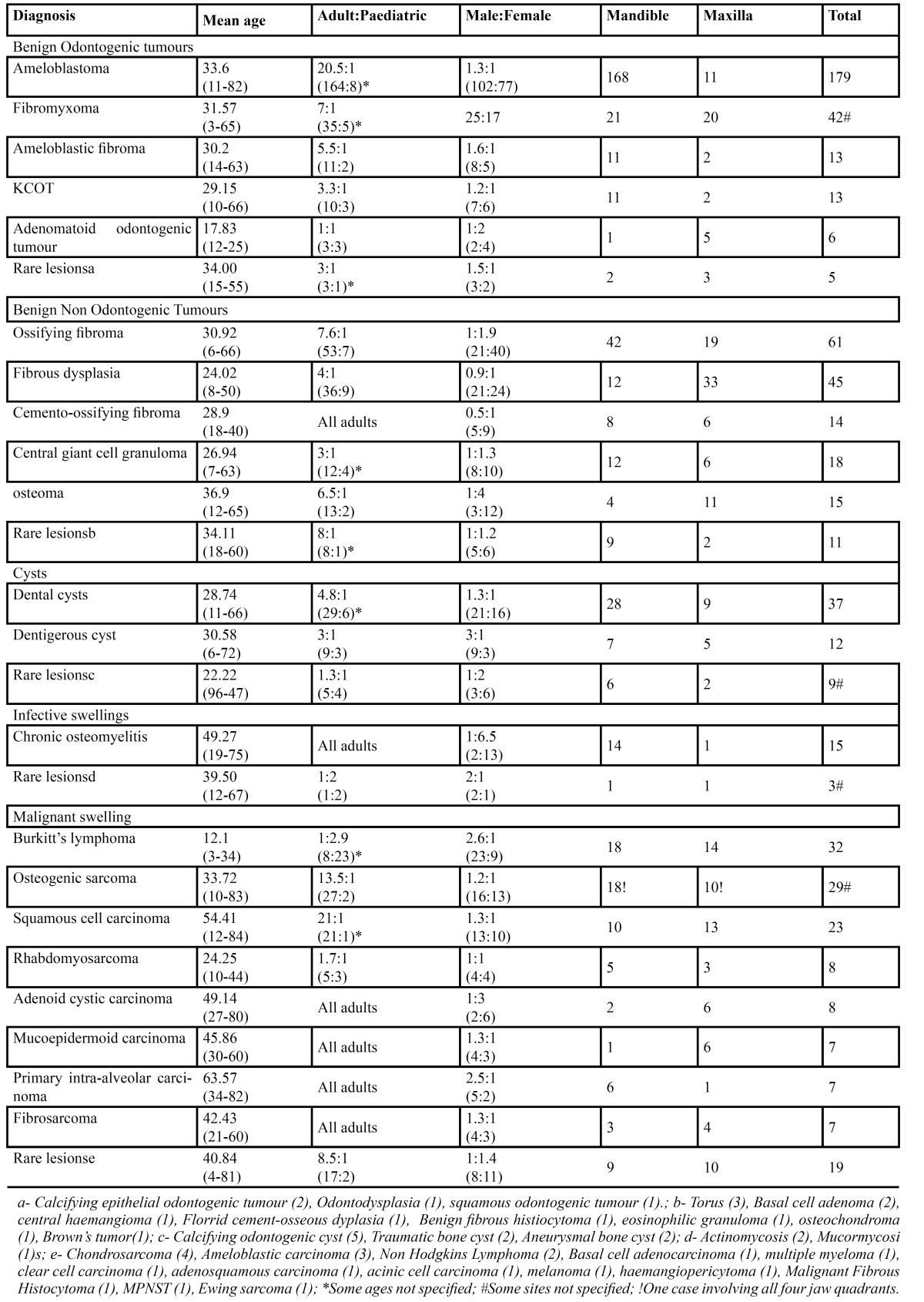
Clinical and histopathological distribution of jaw swellings.

All OT were commoner in adults except for adenomatoid odontogenic tumor (AOT) that was more in the paediatric population ( Table 1). OT formed 45% of all adult jaw swelling while it formed 25.2% in children and adolescents. Ameloblastoma was the commonest OT followed by fibromyxoma. Also, most OT were predominant in males except AOT that was more in females ( Table 1).

The most common NOT was ossifying fibroma (OF) followed by fibrous dysplasia (FD) and the least was osteoma ( Table 1). All tumors categorized as NOT were commoner in females and predominant in adults. NOT comprised 27.1% of all adult jaw swellings while this formed only 22.5% of jaw swellings in children and adolescents. The adult: paediatric ratio was highest for FD, implying that the commonest NOT in children was FD ( Table 1).

Dental cysts (apical and lateral periodontal cysts) large enough to cause a jaw swelling were included in the study and constituted the largest group of cysts ( Table 1). Dental cysts were at least 3 times more in the mandible than the maxilla. The only other common cystic swelling was dentigerous cysts ( Table 1).

Chronic osteomyelitis of the jaws was about 6 times commoner in adult females than males and mostly involved the mandible ( Table 1).The most common malignant jaw swelling was Burkitts’ lymphoma (BL) that was almost 7 times more in children than adults ( Table 1). Osteogenic sarcoma (OS) was the most common malignancy in adults ( Table 1).

## Discussion

Swelling is a sign of inflammatory, cystic and neoplastic disorders of the jaw caused by hypertrophy, hyperplasia, neoplasia and pooling of fluids (4). Jaw swellings in our study were slightly more in males with a male: female ratio of 1.1:1. This suggests that if the varied aetiologies of jaw swellings are not considered exclusively, jaw swellings generally may have little or no gender bias. In a study on general paediatric jaw swellings however, male (55.8%) were affected more than female (44.2%) (5). Thus gender consideration on the platform of age groups may still be significant. The overall mean age in our study was within the fourth decade (32.36 ± 17.14 years). Another study (6) also reported a mean age in the fourth decade almost similar (29.5 years) to ours. They also reported dentigerous cyst as the most common jaw mass, followed by ameloblastomas in their report (6). It is our deduction that they reported a smaller population of jaw swelling cases over a 5-year period. We must therefore logically consider the bio-composition of the population under study in this type of evaluation before making general conclusions.

OT expectedly should be common jaw swellings because the jaw is a reservoir for odontogenic vestigial tissue that could simply undergo neoplastic transformation. This was however not the case in our study as we recorded more NOTs (46.2%) than OTs (41.7%). We have no clear explanation for this outcome. Among OT, Ameloblastoma constituted about 70% in this study. Ameloblastoma is a benign, locally aggressive, infiltrative tumor (7) that is the most common odontogenic tumor in Africa (8,9) , especially in West Africa (10). A study by Arotiba et al. (7) from Ibadan 15 years before this present study found that ameloblastoma formed 59% of all OT seen. From our study it is evident that over the years it has gained yet more prevalence amongst OT. OT, including ameloblastoma, are relatively rare in Caucasians (11).

Adenomatoid odontogenic tumor (AOT) was commoner in females and occurred mostly in the maxilla in kee-ping with another 15-year review of all AOT reports in the PubMed database (12). However unlike this review in which the mean age was 13.2 years (12), we obtained a mean age of 17.8 years and adduce this to probable late presentation of patients in our environment.

We found that fibromyxomas were more in males and had a slight mandibular presentation, this contrasts with others who reported a slight female dominance and a slightly predominant maxillary presentation (13).

NOT are oral tumors that do not arise from the dental structures. They are categorized into tumors of epithelial origin and mesenchymal origin. The most common NOT was ossifying fibroma (OF) followed by fibrous dysplasia (FD) and the least was osteoma. This is in agreement with a study by Toyosawa et al. from Japan (14). The adult: paediatric ratio was highest for FD, implying that the commonest NOT in children was FD.

Literature show that jaw central giant cell granuloma occurs generally before the age of 30 years and that almost 70% occurs in the mandible (15,16,17), we found the mean age of occurrence to be 26.9 years and that mandibular occurrence doubled that of the maxilla.

Dental cysts (apical and lateral periodontal cysts) large enough to cause a jaw swelling were included in the study and constituted the largest group of cysts. This is in agreement with a study by Lawoyin (5), in which periapical cysts formed 12.1% of all jaw cysts. In developing countries seeking proper medical or dental care is expensive and often a last resort after trying other options of management like visiting a traditional healer (18), the use of herbs (18) or self-medication (19). Thus dental infective conditions attain chronicity (5) and the chance of an inflammatory cyst resulting increases. In our study dental cysts were at least 3 times more in the mandible than the maxilla. This does not suggest that caries (which typically precedes inflammatory dental cysts) occurred more in the mandibular than maxillary teeth. A study by Demirci et al. (20) actually found that the maxillary teeth were about twice as carious as mandibular teeth. The only other common cystic swelling was dentigerous cysts. Dentigerous cyst is known as the most common type of non-inflammatory odontogenic cyst (21) and this is in agreement with our finding also.

Typically inflammatory swellings are expected to be the most common type of jaw masses that are caused by mechanical and chemical trauma, radiation injury, infections and immunological mechanisms. In our study however they formed only about 3% of all jaw swellings. Our study has few cases because those included in the study were only those that had surgical intervention and hence had biopsy reports. Most cases would have been managed with medication on out patient basis. Chronic osteomyelitis of the jaws was about 6 times commoner in adult females than males and mostly involved the mandible. We suggest that the high female predominance in our study may imply delayed dental care in female patients due to sociocultural dependence of the females on their husbands for consent and financial support for treatment in our environment. An earlier Nigerian study on jaw osteomyelitis had reported that all patients generally presented late in the natural history of the condition (22). The adult predilection and mandibular involvement is in agreement with a report by Yeoh et al. (23). The higher mandibular involvement may be due to the comparative less medullary bone and consequently less blood supply in the mandible, thus a periapical infective process may find it more conducive to progress to chronic osteomyelitis in the mandible than the spongier maxilla.

The most common malignant jaw swelling was Burkitts’ lymphoma (BL) that was almost 7 times more in children than adults. This is in agreement with a study in Ife, Nigeria by Amusa et al. that also reported lymphomas as the most predominant childhood head and neck malignancy (24). In our study osteogenic sarcoma (OS) was the most common malignancy in adults. Adeyemi et al. (25) from the same center however reported rhabdomyosarcoma (44.2%) as the commonest head and neck sarcoma followed by osteogenic sarcoma (27.3%). Their result varies from ours because of inclusion of additional data from other departments in the hospital. Of all sarcoma types found in the body between 15-20% are diagnosed in the head and neck region but compared to the carcinomas and other jaw malignancies, sarcomas of the jaws are relatively rare (26). The mean age for osteogenic sarcoma in our study was 33.72 years and is closely related to the reports by Ogunlewe et al. (27) from Lagos, Nigeria and Nthumba from Kenya. This indicates that most sarcomas occurred in the third decade. In their studies osteogenic sarcoma was predominant and this is assumed to have influenced the overall age of head and neck sarcomas generally, since osteogenic sarcoma peaks in the third decade of life.

In conclusion, jaw swellings are extensively varied in types and pattern of occurrence. This study has tried to categorize jaw swellings in a simple but comprehensive fashion to allow for easy referencing in local, regional and international data acquisition and epidemiological comparison.
